# Aggressive idiopathic condylar reabsorption after orthognathic surgery: A complex Imaging diagnosis

**DOI:** 10.1016/j.radcr.2025.02.098

**Published:** 2025-03-23

**Authors:** Bruno Nifossi Prado, Lucas Cavalieri Pereira, Juliana NIfossi Prado

**Affiliations:** a São Leopoldo Mandic University, Campinas, São Paulo, Brazil; b Private Clinic, São Paulo, São Paulo, Brazil

**Keywords:** Idiopathic condylar resorption, Condylar resorptions, Temporomandibular joint, Orthognathic surgery

## Abstract

Idiopathic condylar resorption (ICR) is a pathological condition of unknown origin that affects the temporomandibular joint (TMJ) and can lead to malocclusion, facial asymmetry, TMJ dysfunction and orofacial pain. Imaging examinations are essential for diagnosis and planning of future treatment. To diagnose ICR, it will always be necessary to perform imaging tests and associate them with predisposing risk factors to understand the level of bone remodeling and mechanical trauma performed to propose the best type of treatment. The objective of this study was to elucidate the clinical case of a patient who underwent orthodontics and orthognathic surgery and after 2 years, occlusion recurrence was diagnosed with a severe and active ICR. In addition, it demonstrates the importance of requesting postoperative imaging examinations and clinical radiographic monitoring.

## Introduction

Idiopathic condylar resorption (ICR) is a pathological condition of unknown origin that affects the temporomandibular joint (TMJ) and can lead to malocclusion, facial asymmetry, TMJ dysfunction and orofacial pain [1,2]. This resorption of the mandibular condyles can lead to a loss of posterior vertical height of the mandible, rotating and producing an anterior open bite, which is associated with mandibular retrognathia [3,4].

There are some factors that lead to ICR predisposition, the first of which is the female sex, approximately 9:1 female to male; the age from 10 to 40 years old, but with a predominance in the puberty phase; the angle class II relationship with or without anterior open bite; and dolichocephalic patients with a high occlusal plane and mandibular plane [1,2,5].

The etiology is still unknown and can be classified according to the severity of joint degeneration. Osteoarthritis, osteoarthrosis and arthrosis are possible causes of condylar remodeling. Other nomenclatures include avascular necrosis, osteonecrosis, condylar atrophy, and condylar osteolysis [3,6,7].

During mandibular orthognathic surgery, mandibular advancement tends to move the condyle to a more anterior position in the mandibular fossa, compressing the articular disc and mandibular bone. When this occurs, natural adaptation tends to occur in the TMJ, however this adaptation capacity is exceeded, and the mandibular condyle undergoes remodeling [8].

Treatment is individualized and depends on the degree of TMJ involvement and the associated symptoms. Clinical treatments, such as analgesia, medications, physical therapy and occlusal splints are generally indicated in early cases of ICR. In cases where the anatomy of the TMJ is compromised, surgical procedures such as TMJ arthroscopic surgery, discopexy, orthognathic surgery, or total TMJ prosthesis are performed [9].

## Case report

A 24-year-old female patient complained of bilateral preauricular pain, otalgia and severe headaches. She complained that the bite was open, but she had already undergone orthognathic surgery 2 years ago to correct this complaint with another surgeon. According to the patient, her bite returned to its original position before orthognathic surgery in addition to constant pain. After seeking an explanation of her constant pain and the recurrence of her bite after orthognathic surgery, the patient came to us for evaluation, diagnosis, and treatment.

In her medical history, the patient had no systemic disease, denied a history of allergies, and reported that she had used contraceptives for 5 consecutive years. As she experienced pain in the temporomandibular joint, analgesics (ibuprofen 400 mg/1 tablet every 6 hours) and nonsteroidal anti-inflammatory drugs (Meloxican 15 mg/1 tablet per day) were used monthly. There was no treatment for temporomandibular disorders or retrognathia prior to orthognathic surgery. The symptoms she reported before orthognathic surgery (facial pain and breathing difficulty) persisted after the surgical procedure. The patient reported that before the orthognathic surgery, she underwent presurgical orthodontic treatment, and her facial pain was always present during this period.

Clinical examination revealed bilateral joint pain in the TMJ, difficulty in opening the mouth, bite deviation to the right side, mandibular retrognathia and anterior open bite. There have also been reports of nocturnal bruxism being associated with anxiety. With a history of orthognathic surgery and nocturnal bruxism, we investigated the possible causes of this pain and for such a large recurrence in occlusion. Imaging tests were performed to diagnose possible bone or dental pathologies.

The first exams to be requested were X-rays (Orthopantomography and Lateral radiograph), as they are quick and cheap exams. Based on the results of the radiographs and possible anatomical changes in the mandibular condyle, we requested a radionuclide bone scan, exams to evaluate the metabolic activity in the temporomandibular joint, an Magnetic Resonance Imaging (MRI) to evaluate the condition of the soft tissues and articular discs and a Computed Tomography (CT) to evaluate the characteristics nones of this joint.

The correct diagnosis of ICR is only possible after evaluating complementary tests, which gives us aa parameter of how the patient is currently correlating with her symptoms and the possible steps to be taken in the future.

Owing to the aggressiveness and speed of this reabsorption, analgesic measures were taken, and local measures with the application of high-viscosity hyaluronic acid to improve local mobility and subsequently the planning of a new orthognathic surgery with total TMJ prostheses will be carried out.

### Orthopantomography and lateral radiograph

**Fig. 1:**
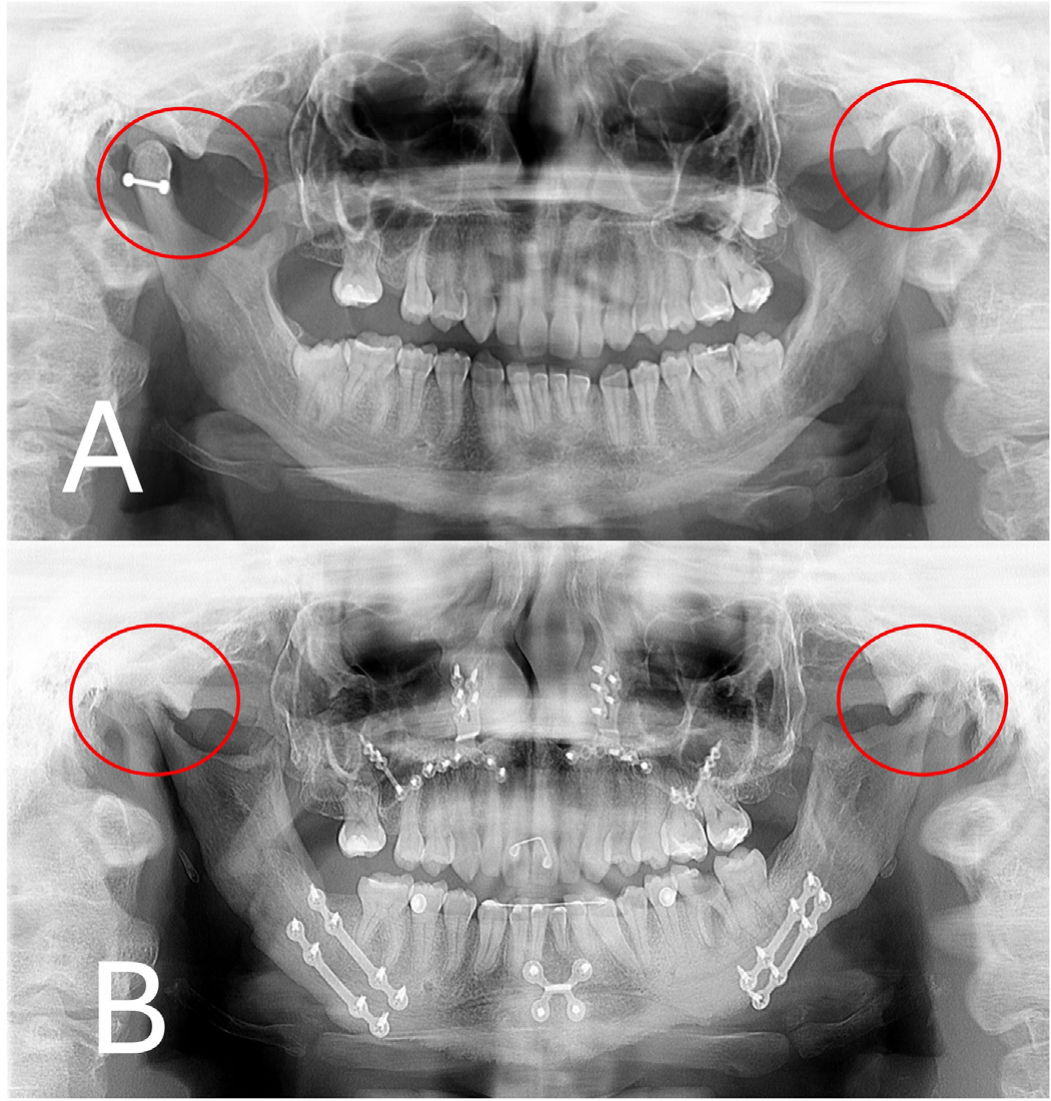
Orthopantomography (A) before orthognathic surgery. Normal condylar process and (B) 2 years after the surgical procedure with condylar process thin and resorbed.

**Fig. 2:**
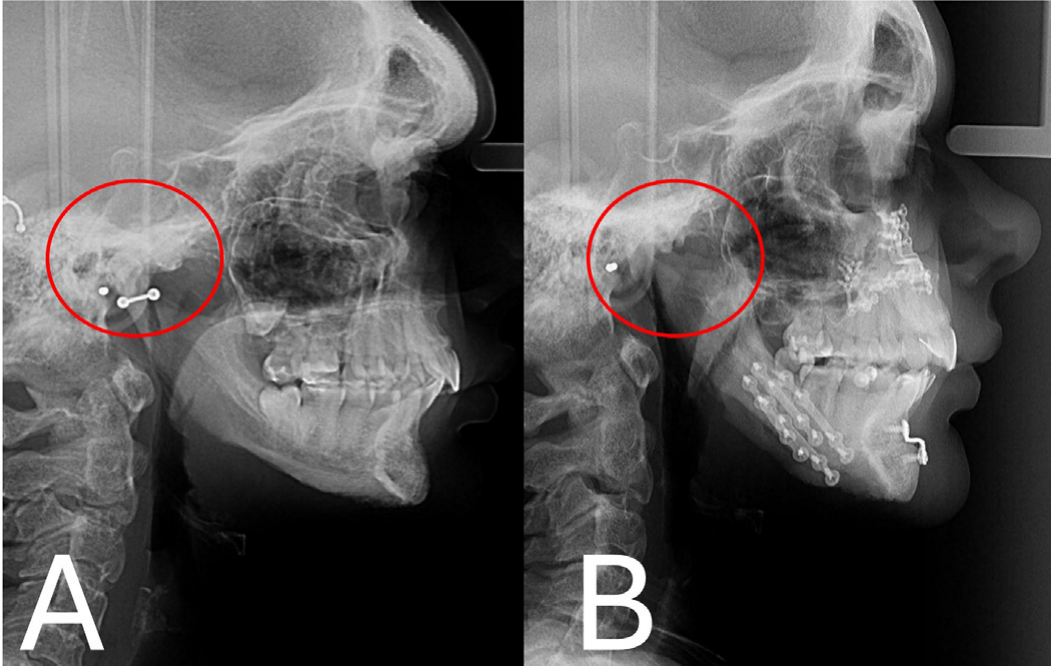
Lateral radiograph (A) before orthognathic surgery and (B) 2 years after surgery showed the occlusal relapse and retrognathia.

On orthopantomography, we can visualize condylar thinning and loss of bone volume even in a bidimensional form, especially when compared with an X-ray before surgery (Fig. 1). On lateral radiography, the relapse of the mandible with retrognathia is evident, indicating loss of posterior height of the mandibular condyle (Fig. 2).

### Radionuclide bone scan

**Fig. 3:**
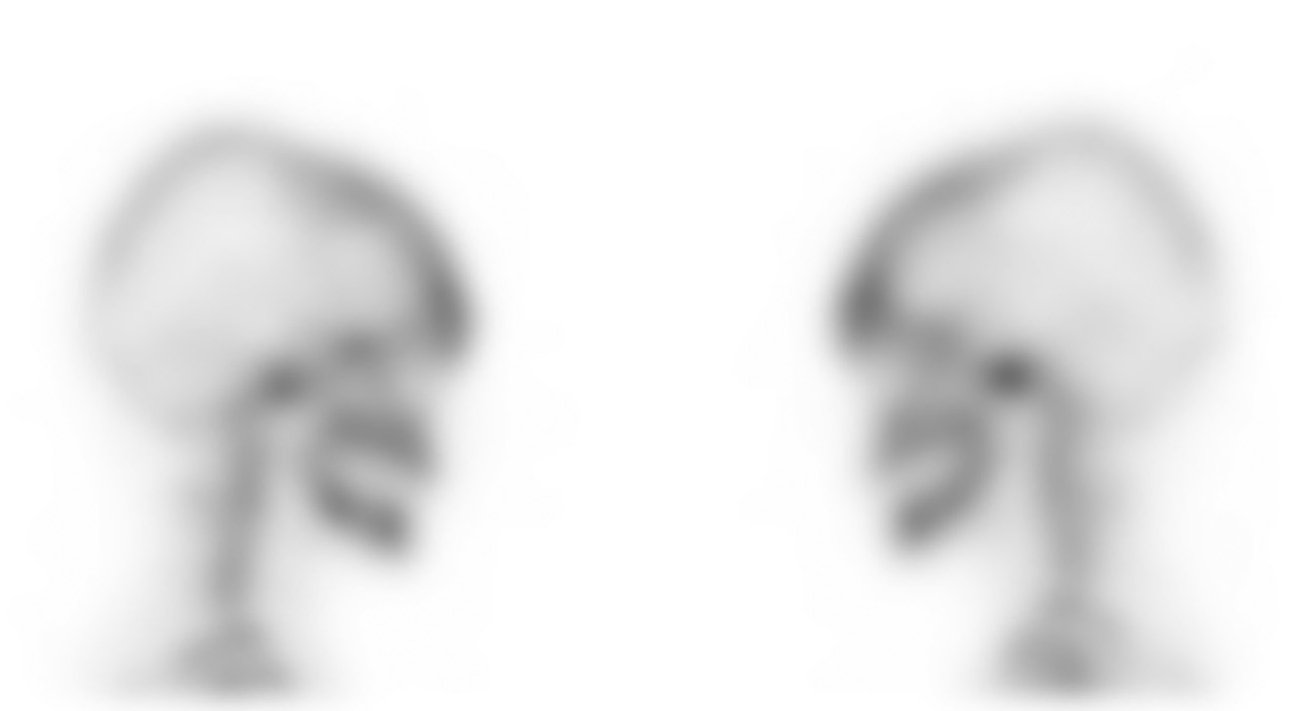
Radionuclide bone scan 2 years post orthognathic surgery showed hyper uptake of the radiopharmaceutical in the TMJ joints.

The results of bone scintigraphy using MDP^_99m^ Tc showed hyperuptake of the radiopharmaceutical in the TMJ joints, which was more evident on the left side, and an apparent reduction in joint space was also noted (Fig. 3).

### Magnetic resonance imaging (MRI)

The Condylar processes of the mandible, mandibular fossa, and articular tubercle showed irregularities and straightening of their articular surfaces with reduction of disc spaces.

**Fig. 4:**
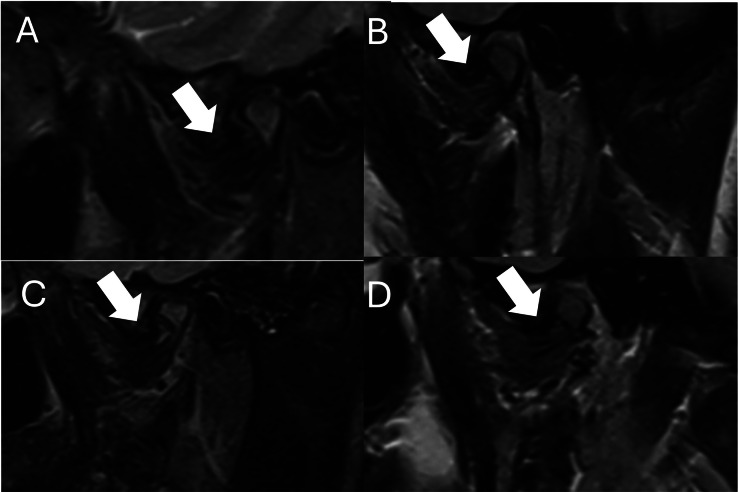
MRI T2 Weighted Imaged. Sagittal view. Arrows indicate disk displacement (A) Right side close mouth. (B) Right side open mouth. (C) Left side close mouth and (D) Left side open mouth.

Articular discs were displaced anteriorly, with preserved morphology and signal intensity, without signs of recapture after dynamic mouth opening maneuvers. There was no evidence of joint effusion, preserved condylar transition movements, or unaltered lateral pterygopalatine muscles (Fig. 4).

### Computed tomography

Using computed tomography (CT), with 0.5mm thick slices without contrast, a severe erosive process was visualized in the mandibular condyles with loss of bone height and thickness. Flattening was noted in the mandibular fossa, with local bone rarefaction and loss of convexity.

**Fig. 5:**
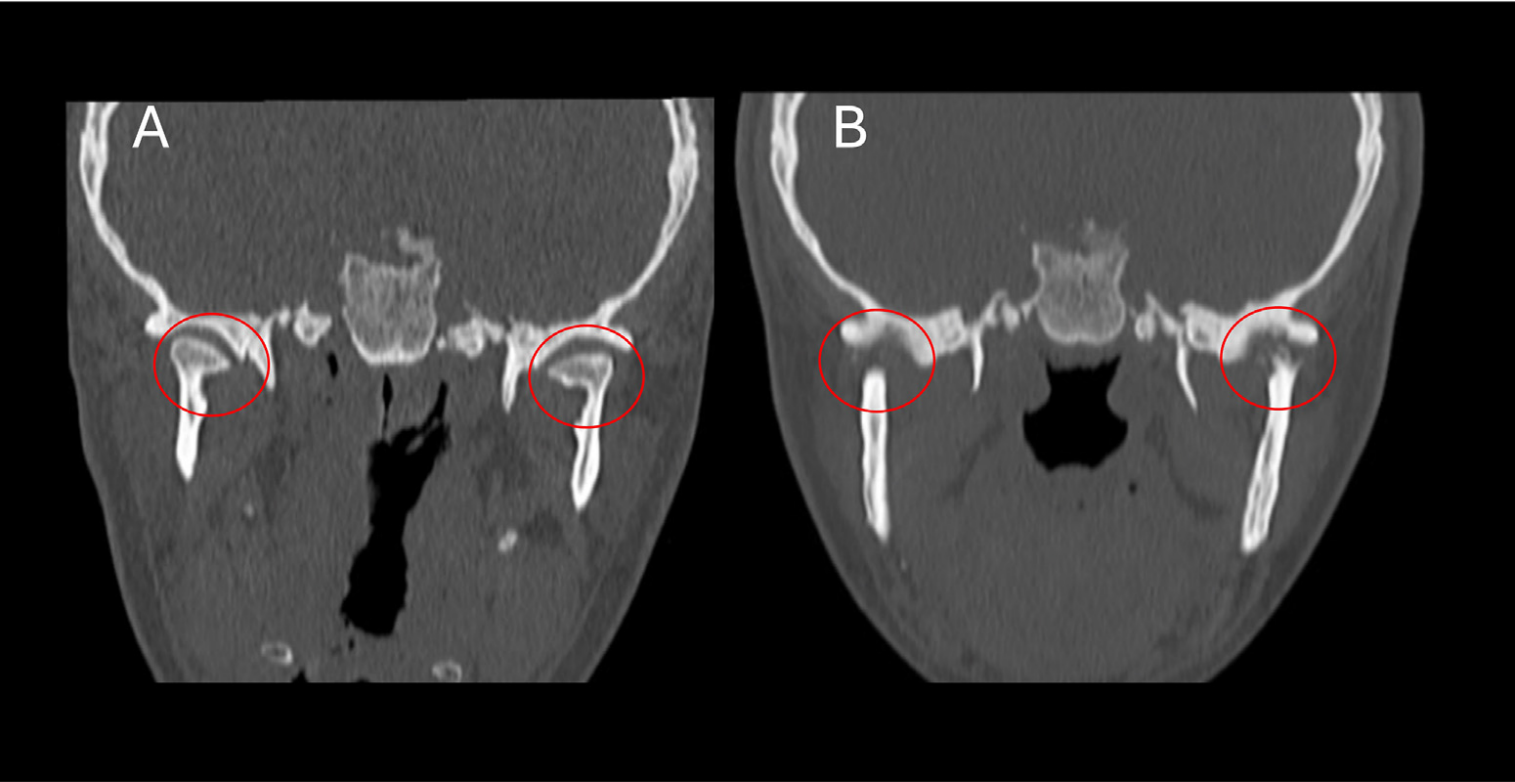
CT Image of TMJ. Coronal view (A) Before orthognathic surgery. Normal condylar process and (B) 2 years after procedure incredible loss of bone volume in the heads of mandible and flattening of mandibular fossa.

**Fig. 6:**
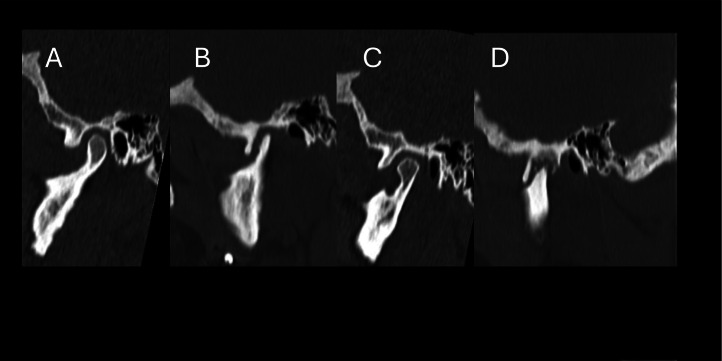
CT Image of TMJ. Sagittal view. (A) Right condyle before surgery. Normal condylar process (B) Right condyle 2 years after surgery. Incredible loss of bone volume in the heads of mandible and flattening of mandibular fossa (C) Left condyle before surgery. Normal condylar process and (D) Left condyle 2 years after surgery, incredible loss of bone volume in the heads of mandible and flattening of mandibular fossa.

Comparing the CT before surgery and 2 years postoperatively, with the ICR already installed, in the coronal section it was possible to observe the incredible loss of bone volume in the mandibular heads and flattening of the mandibular fossa (Fig. 5). In sagittal sections, the imagens demonstrated thinning of the condylar process, intense bone resorption with loss of joint space and loss of convexity of the temporomandibular joint (Fig. 6).

## Discussion

The predilection for the female sex is evident in all studies, associated with predisposing factors age, retrognathia, class II occlusion, dolichocephalies are a perfect match for ICR^1^. It is also reported as a cheerleader syndrome associated with all these characteristics and the trauma faced suffered by these athletes during training [5]. In a study comparing young female patients undergoing orthognathic surgery who were class II and suffered ICR with class II patients who did not undergo ICR, nonsurgical factors were predominant, such as posterior inclination of the condylar neck, a high mandibular plane angle, and a short posterior facial height ratio. Recommending that patients with these characteristics are aware of the surgical risk of ICR [10].

Hormonal aspects also come into play; in studies with rats it was demonstrated that estradiol deficiency associated with TMJ overload produces morphological changes in the mandibular condyle and changes in bone microstructure in areas of poorer bone [11] Blood analysis of 16 patients with ICR in 12 of them showed low levels of estradiol compared to Korean women of the same age group. Five of the 12 patients were diagnosed with osteopenia using bone densitometry. The extent of mandibular advancement was also statistically significant with a tendency towards recurrence [12].

Bone remodeling in ICR is based on an individual's adaptive capacity and mechanical stimuli. Regardless of the cause, functional remodeling is characterized by the adaptation of the TMJ structures in response to mechanical factors. Factors such as age, hormones and systemic diseases can contribute to TMJ bone remodeling, especially if biomechanical forces can alter adaptive remodeling capacity [7,13].

In orthognathic surgeries, when fixing mandibular osteotomy mainly with bicortical screws, lateral or medial torque of the condyle in the mandibular bone may occur. If there is no adequate adaptation occlusal stability associated with internal degenerate, parafunction, microtrauma, or occlusal instability, it can lead to severe bone remodeling [7,13].

A study evaluating condylar position in class III patients with CT over a long period, found that forward movements in the maxilla and mandible are more protective of the condyles, presenting little loss of bone volume [14]. This goes against the results of studies with class II and counterclockwise rotating movements [8].

When it comes to treating ICR, we can eradicate etiological factors, stabilize unstable occlusion, and correct the resulting occlusal deformity [7]. Another way to treat it is to perform orthognathic surgery again with disc repositioning and removal of the hyperplastic synovial tissue [1]. Total TMJ prostheses can be used when bone destruction is extensive with loss of adjacent tissues [6], and clinical treatment with orthodontics alone or physiotherapy is ineffective in such cases [9].

Pharmacological treatment to control the ICR involves many steps The use of antioxidants, ômega-3 fatty acids, tetracyclines, nonsteroidal anti-inflammatories and inflammatory cytokine inhibitors to aid in preventing and controlling ICR [15]. After bone stabilization control, orthognathic surgery with counterclockwise rotation is becomes necessary to restore occlusal stability [15].

Treatments with total TMJ prostheses have become efficient mainly concomitantly with orthognathic surgery. Anatomical replacement of the TMJ has proven to be efficient in terms of pain score, TMJ sounds, mouth opening and dietary restriction [16,17].

Radiographic examinations such as orthopantography and lateral radiography are the initial examinations used to understand this problem. CT scans ere used to determine the extent of the affected bone. MRI we can be used to determine how the TMJ and its structures are affected. Radionuclide bone scans showed that this degeneration was still active. All examinations are important and should be performed in these complex cases.

In summary, to diagnose ICR it will always be necessary to perform imaging tests and associate them with predisposing risk factors to understand the level of bone remodeling and mechanical trauma performed to propose the best type of treatment. It is very important for patients undergoing orthognathic surgery to have a long-term clinical radiographic follow-up, especially in patients with predisposing factors.

## Ethical approval

This study was conducted in accordance with the Declaration of Helsinki. Informed consent was obtained from all participants involved in the study.

## Patient consent

I confirm that I have obtained the patient's consent for the publication of this article “Aggressive idiopathic condylar reabsorption after orthognathic surgery: A complex Imaging diagnosis”.
